# Correlation among irrational parenthood cognitions, fertility stress, and social support in patients with repeated implantation failure and the mediating effect of fertility stress: a cross-sectional survey

**DOI:** 10.1007/s10815-023-02953-2

**Published:** 2023-11-03

**Authors:** Yan Lvy, Fang Zhang, Zhongbo Cai, Danting Zhong, Lanfeng Xing

**Affiliations:** Department of Reproductive Endocrinology, School of Medicine, Women’s Hospital, Zhejiang University, Hangzhou 310000 Zhejiang, Province, People’s Republic of China

**Keywords:** Repeated implantation failure, Fertility cognition, Fertility stress, Social support, Mediating effect

## Abstract

**Purpose:**

This study investigated the relationships among irrational conceptions of parenthood, fertility stress, and social support, as well as the mediating effect of fertility stress, in patients with repeated implantation failure.

**Methods:**

Patients who underwent assisted reproductive technology due to repeated implantation failure at the Reproductive Centre at Women's Hospital between January 2020 and August 2022 were selected using cross-sectional research and convenience sampling. A total of 129 patients with recurrent implantation failure were investigated using the Irrational Parenthood Cognitions Questionnaire (IPCQ), Fertility Problem Inventory (FPI), and Social Support Rating Scale (SSRS).

**Results:**

The differences in irrational parenthood cognitions, fertility, stress, and social support among patients with repeated implantation failure in the education and yearly household income subgroups were statistically significant (*P*<0.001). Pearson correlation analysis revealed that irrational parenthood cognitions were favorably correlated with all measures of reproductive stress (*r*=0.384 to 0.664, all *P*<0.01) and negatively correlated with social support (*r*=-0.310, *p*<0.01). The fertility stress of patients with repeated implantation failure fit the structural equation model of irrational parenthood cognitions and social support well [X2/df=2.04, Comparative Fit Index (CFI)=0.944, Tucker–Lewis (TLI)=0.905, Root Mean Squared Error of Approximation (RMSEA)=0.090]. The bootstrap test results revealed that the mediating effect of the 95% *CI* ranged between -0.506 and -0.109, and the interval did not contain 0. Fertility stress had a strong mediating effect on the relationship between irrational parenthood cognitions and social support.

**Conclusions:**

The mediating effect of reproductive stress on the relationship between irrational parenthood cognitions and social support in patients with repeated implantation failure was significant. It is important for medical and nursing staff to address physical and psychological illnesses and develop effective intervention strategies from the perspectives of fertility stress, social support, and cultural background, with the ultimate goal of improving mental health.

## Introduction

In China, on average, one in eight couples experiences infertility, and the number of patients undergoing treatment with assisted reproductive technology, the primary method of fertility therapy, is increasing annually [1, 2]. Assisted reproductive technology is used to meet the fertility demands of numerous families; however, it must be acknowledged that even with the most advanced in vitro fertilization (IVF) technology, patients still experience repeated failures. According to data, up to 5 to 10% of patients who have undergone at least two fresh cycles or freeze–thaw cycles with a total of no less than four good-quality embryos or two blastocysts transferred without clinical pregnancy experience repeated implantation failure (RIF) [3, 4]. Irrational parenthood cognition is a 15-year-old term that refers to a cognitive bias toward fertility in which people believe that they must have a child to have a happy life [5]. Children provide many benefits to couples and communities: They can stabilize or secure marital relationships, increase and strengthen kinship ties, validate individual roles, offer spiritual comfort or religious redemption, provide financial benefits, ensure immortality, provide a legacy for property or values, and develop unique interpersonal relationships. Infertility can thus have many psychosocial consequences with cultural, religious, and social ramifications for both men and women [6]. Although the value and importance of children may be universal, the meaning of having children, and thus childlessness, may vary across cultures [7]. Due to the influence of traditional Confucianism in China, women with infertility or other reasons for not having children frequently experience psychologically degrading thoughts, such as low self-esteem, loneliness, and shame regarding their families, and may see their inability to have children as a strain on their lives. These illogical views about fertility might cause women experiencing infertility to feel different and unaccepted, resulting in negative emotions and stress [8–10]. However, by acting on the hypothalamic-pituitary-adrenal (HPA) axis, negative emotions can decrease the release of sex hormones and cause abnormalities in reproductive organ function [11]. Fertility stress is a major research topic in reproductive medicine, and infertility is considered a stigmatizing and isolating experience for patients because it is a chronic stressor with minimal controllability. The lengthy and arduous treatment, extensive medical costs, invasive procedures, family and social pressures during the assisted conception period, and especially the failure of assisted conception can cause varying degrees of physical and psychological stress in patients experiencing infertility [12, 13]. Consequently, infertility patients require substantial support from their partners, families, and society. Social support plays a vital role in the stress response process, reducing a patient's discomfort caused by sickness while also improving their poor psychological condition and enhancing their self-confidence in the treatment [14]. The present study examined the characteristics of irrational parenthood cognitions, fertility stress, and social support in patients with RIF, as well as the mediating effect of fertility stress, to provide theoretical guidance for clinical psychological interventions for patients with RIF.

## Materials and methods

Patients undergoing assisted reproductive technology for RIF at the Reproductive Centre at Women's Hospital, School of Medicine, Zhejiang University in Hangzhou, People’s Republic of China, from January 2020 to August 2022 were selected using a cross-sectional and convenience sampling method. Using the Kendall sample size estimation method [15], the sample content was calculated to be five–ten times the number of independent variables, and the sample size was increased by ten percent to calculate a sample size of 61–122 patients, considering the possibility of invalid questionnaires. The following inclusion criteria were used: (i) patients who met the diagnostic criteria for RIF proposed by Polanski et al. [3]; and (ii) patients who were informed about the study, volunteered to participate, and signed an informed consent form.

The Institutional Review Board of Women’s Hospital, School of Medicine, Zhejiang Hospital authorized this research (Approval Number: IRB-20220124-R).

Participants were asked to complete a standard questionnaire that inquired about their age, place of residence, duration of marriage, degree of education, family income, marital status, the existence of biological children, source of birth pressure, number of transfers, etc.

The Irrational Parenthood Cognitions Questionnaire (IPCQ) was developed by Fkkes et al. in the Netherlands and translated into Chinese by Li Jianan from Nanjing University in 2016 [16]. This questionnaire was designed to assess conceptions of fertility among women of reproductive age. The IPCQ consists of 14 questions scored using a Likert-type scale, with a total possible score ranging from 14 to 70; a score of 42 indicates a moderate level of understanding. A higher rating implies that the responder anticipates that having a child would lead to happiness. The Cronbach's alpha coefficient for the English version of the scale was 0.87.

The Fertility Problem Inventory (FPI) was created by Canadian researchers Newton et al. and translated by Peng [17]. The scale consists of 46 items and five subscales: the societal concern, sexual concern, relationship concern, rejection of childfree lifestyle, and need for parenthood subscales. Each item is assessed on a 6-point Likert scale, ranging from 1 (I disagree) to 6 (I entirely agree). The Cronbach’s alpha coefficients range from 0.77–0.93 for the FPI total score and subscale scores, indicating strong reliability and validity. Owing to its applicability and comprehensiveness, the FPI has been widely utilized in international infertility investigations. The Chinese version has been validated as having excellent reliability and validity and is appropriate for the clinical evaluation of infertility patients in China.

The Social Support Rating Scale (SSRS) is a self-assessment scale established by Xiao Shuiyuan [18] to evaluate the social support status of patients experiencing infertility. It consists of ten items encompassing three dimensions: objective assistance, subjective assistance, and social support use. The total scale score is the sum of the scores of the 10 items (64 points), with higher scores indicating higher levels of social support.

Before the survey, the researchers provided training for each investigator. The investigators collected patient data on the day of admission and introduced the requirements for completing the questionnaire using uniform instructional language; individuals with reading difficulties were assisted in completing the questionnaire. Individual questionnaires were collected; questionnaires with consistent answers and those without answers for more than two-thirds of the questions were deemed illegitimate and excluded from the analysis. In this study, 140 questionnaires were distributed, of which 138 were collected, and 129 valid questionnaires were returned, with a valid return rate of 93.48%.

### Statistical analysis

SPSS (version 26.0) was used for statistical analysis of the data. Count information is reported as proportions and percentages (%). The measurement data conforming to a normal distribution are expressed as the mean±standard deviation (mean ±SD), and groups were compared using a t test or one-way ANOVA. Pearson correlation analysis was used for correlation analysis. This study used Mplus 8.3 software to create a structural equation model plot; the model was corrected by applying the bias-corrected nonparametric percentile bootstrap method with 1000 random bootstrap samples from the original data, and 95% confidence intervals (*CIs*) were calculated to test the mediating effect of fertility stress on the relationship between irrational parenthood cognitions and social support. Differences were considered statistically significant at *P*<0.05.

## Results

### Demographic characteristics

General information was included in the univariate analysis, in which irrational parenthood cognitions, fertility stress, and social support were included as independent factors. Differences in irrational parenthood cognitions were statistically significant (*P*<0.001) in the subgroups of education, annual family income, relationship with one’s partner, and social pressure from others; differences in fertility stress were statistically significant (*P*< 0.001) in the subgroups of education, annual family income, and social pressure from others. For the subgroups of literacy, annual household income, connection with one’s partner and social pressure from others, the differences in social support were statistically significant (*P*<0.001). The results are further illustrated in Table 1.

**Table 1:** Comparison of irrational parenthood cognitions, fertility stress and levels of social support among RIF patients with different demographic characteristics (*n*=129)

Variable	Group	Cases(%)	IPCQ	*P* Value	FPI	*P* Value	SRSS	*P* Value
Age	≤30y	26 (20.2)	43.46±9.99	0.125	163.38±27.91	0.468	32.65±5.16	0.440
	31-35y	49 (38.0)	39.92±10.91		158.90±21.69		34.45±4.74	
	36-40y	40 (31.0)	42.10±8.80		163.75±17.54		33.77±4.86	
	>40y	14 (10.9)	35.79±8.72		166.93±19.72		34.36±7.12	
Residential location	Countryside	37 (28.7)	44.43±11.10	0.191	169.00±22.17	0.025	31.65±4.84	0.006
	Town	86 (66.7)	38.70±8.62		159.65±19.59		34.80±4.82	
	Urban and rural areas	6 ( 4.7)	49.83±12.25		156.33±38.19		34.17±7.86	
Nationalities	Han Chinese	119 (92.2)	40.85±10.20	0.957	161.96±22.21	0.574	33.93±5.25	0.522
	Minority	10 ( 7.8)	41.00±8.10		164.80±14.19		33.10±3.70	
Marital status	First Marriage	105 (81.4)	40.64±10.31	0.564	160.87±21.66	0.152	34.07±5.13	0.371
	Remarriage	24 (18.6)	41.83±8.79		167.92±21.19		33.00±5.22	
Marital age	1-2y	8 ( 6.2)	38.75±7.32	0.572	157.25±16.64	0.914	31.75±5.95	0.947
	2-5y	61 (47.3)	40.69±10.55		162.05±18.26		34.02±5.46	
	6-10y	43 (33.3)	41.33±10.33		165.79±24.59		34.56±5.10	
	>10y	17 (13.2)	41.29±8.93		155.82±26.56		32.59±3.26	
Level of education	Primary and below	28 (21.7)	44.14±8.52	< 0.001	172.14±29.31	< 0.001	32.11±4.92	< 0.001
	Junior	10 ( 7.8)	48.00±9.38		164.60±34.88		32.40±3.89	
	Senior	30 (23.3)	42.90±12.55		164.33±17.25		32.23±5.67	
	Tertiary	44 (34.1)	38.07±7.98		156.30±14.95		35.34±4.69	
	University degree or above	17 (13.2)	34.88±7.52		155.76±12.49		36.71±4.31	
Occupation	Workers	14 (10.9)	43.86±11.82	0.629	159.43±33.25	0.228	32.07±4.55	0.684
	Farmers	2 ( 1.6)	32.00±2.83		140.00±0.00		28.00±4.24	
	Civil Servants	4 ( 3.1)	40.00±11.58		162.75±14.31		37.00±4.97	
	Self-employed	12 ( 9.3)	43.67±10.94		162.33±18.88		34.25±4.94	
	Teachers	9 ( 7.0)	36.44±6.13		156.00±6.63		35.00±4.06	
	Professional and technical staff	19 (14.7)	37.05±5.82		161.16±13.71		37.00±4.47	
	Freelance	23 (17.8)	44.61±10.66		163.87±17.28		32.17±4.25	
	Others	46 (35.7)	40.24±10.26		164.67±25.41		33.63±5.67	
Annual household income	≤5million	16 (12.4)	48.31±8.90	< 0.001	171.88±44.16	< 0.001	29.81±4.35	< 0.001
	5-10million	35 (27.1)	43.51±10.75		168.63±17.36		33.43±5.34	
	10-30million	46 (35.7)	40.85±8.52		160.63±14.74		33.89±4.68	
	>30million	32 (24.8)	34.25±7.91		152.50±12.37		36.34±4.68	
With/without biological children	Yes	16 (12.4)	45.12±8.62	0.051	171.75±19.57	0.052	32.62±5.28	0.325
	No	113 (87.6)	40.26±10.09		160.82±21.69		34.04±5.12	
Sources of fertility stress	Parents-in-law	19 (14.7)	44.53±10.07	0.119	169.37±21.38	0.146	33.00±4.42	0.275
	Parents	10 ( 7.8)	42.00±9.70		166.40±17.19		34.10±5.80	
	Husband	81 (62.8)	40.06±10.04		159.96±18.31		33.65±5.26	
	Myself	13 (10.1)	41.08±10.92		165.23±40.27		35.85±5.61	
	Other relatives	3 ( 2.3)	33.00±3.61		153.33±18.93		36.67±1.53	
	Friends, classmates, colleagues, neighbors	3 ( 2.3)	42.33±8.50		158.00±11.00		33.00±4.36	
Relationship with husband	Very good	71 (55.0)	38.58±8.66	< 0.001	159.85±22.08	0.227	34.73±5.06	< 0.001
	Better	45 (34.9)	41.91±10.45		163.58±16.46		33.69±4.84	
	General	10 ( 7.8)	47.20±10.48		172.20±21.35		31.20±4.49	
	Poorer	1 ( 0.8)	66.00±NA		199.00±NA		22.00±NA	
	Very bad	2 ( 1.6)	54.00±4.24		145.00±80.61		26.50±2.12	
Social pressure from others	Very high	16 (12.4)	50.44±10.81	< 0.001	174.19±27.13	< 0.001	30.81±6.63	0.021
	Higher	34 (26.4)	41.50±10.19		168.53±20.31		33.76±5.11	
	General	60 (46.5)	39.85±8.80		158.08±21.76		34.52±4.78	
	Smaller	9 ( 7.0)	34.00±5.94		154.89±8.55		32.78±3.31	
	Very Small	10 ( 7.8)	35.60±8.04		152.50±9.45		36.20±4.61	

Values are given as the mean ± standard deviation, or frequency (%)

### Correlation analysis

The ratings for irrational fertility perceptions (40.86±10.02), fertility stress (162.18±21.67) and social support (33.87±5.14) were comparable to the national norm (34.56±3.73). According to the results of the Pearson correlation analysis, irrational parenthood cognitions were positively correlated with fertility stress and its dimensions (*r*=0.384–0.664, all *P*<0.01) and negatively correlated with social support (*r*=-0.310, *p*<0.01). The results are depicted in Table 2.

**Table 2:** Correlational analysis of irrational parenthood cognitions, fertility stress and social support in patients with RIF (*n*=129, *r*)

	IPCQ	FPI	Social Concern	Relationship Concern	Need for Parenthood	Rejection of Childfree Lifestyle	Sexual Concern	SSRS	Objective Support	Subjective Support	Social Support Utilization
IPCQ	1										
FPI	0.465*	1									
Social Concern	0.384*	0.831*	1								
Relationship Concern	0.540*	0.861*	0.673*	1							
Need for Parenthood	0.664*	0.744*	0.569*	0.648*	1						
Rejection of Childfree Lifestyle	-0.532*	0.062	-0.032	-0.137	-0.419*	1					
Sexual Concern	0.399*	0.760*	0.537*	0.632*	0.510*	-0.153	1				
SSRS	-0.310*	-0.081	-0.069	-0.201*	-0.132	0.228*	-0.086	1			
Objective Support	-0.344*	-0.158	-0.129	-0.259*	-0.223*	0.260*	-0.151	0.660*	1		
Subjective Support	-0.112	0.040	0.040	-0.057	0.064	0.061	0.007	0.806*	0.187*	1	
Social Support Utilization	-0.277*	-0.116	-0.120	-0.160	-0.235*	0.239*	-0.075	0.631*	0.311*	0.285*	1

*P<0.05，**P<0.01

### Structural equation modeling

**Fig. 1:**
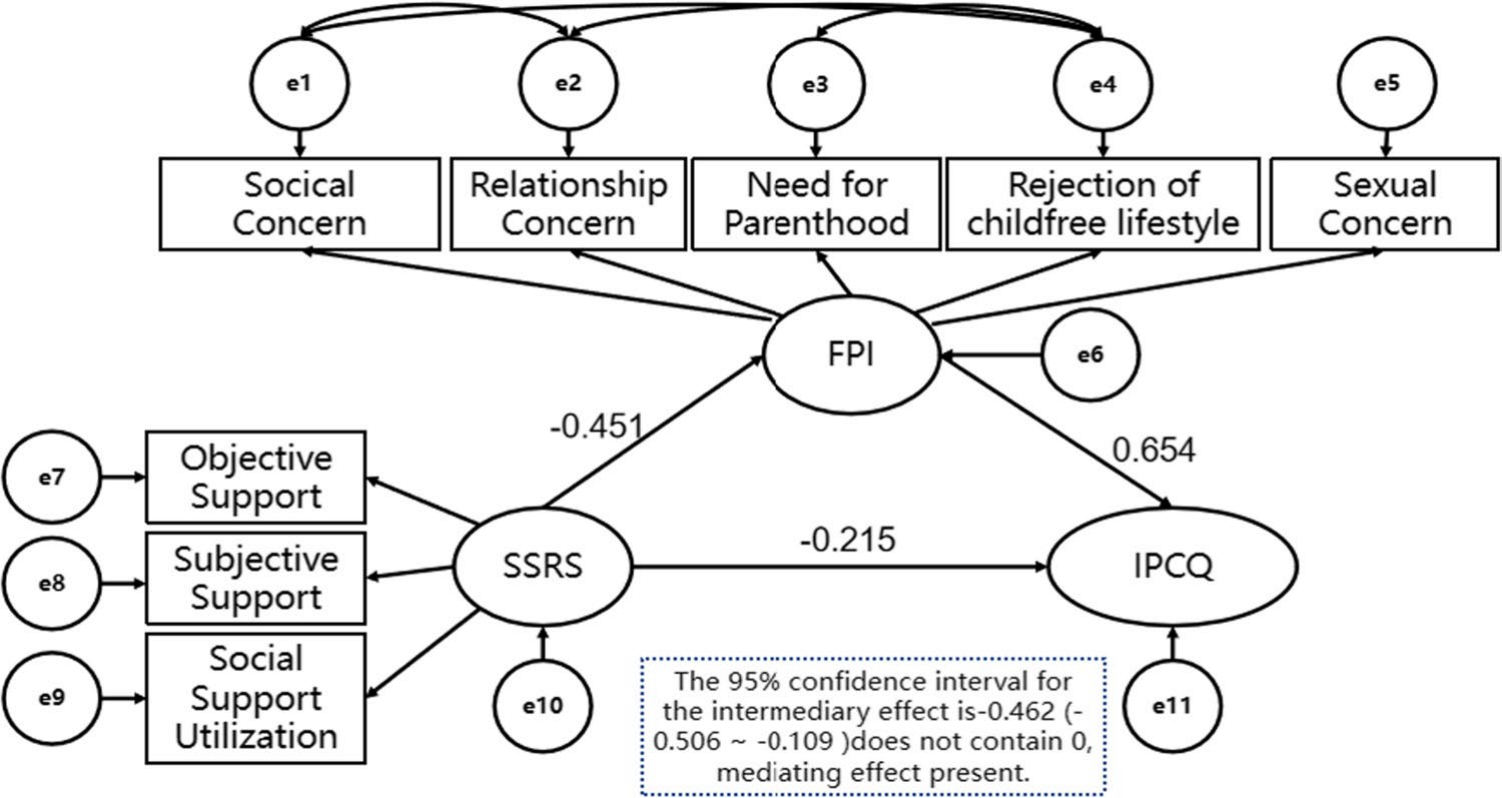
Structural equation modeling of irrational parenthood cognitions, fertility stress, and social support in patients with repeated implantation failure

In this study, the maximum likelihood method was used to model the relationship based on existing theories, and the model was modified according to the modified index (MI) indicator to further explore the relationship among irrational parenthood cognitions, fertility stress, and social support. The following modified model fit indices affirmed that the model had a good fit: X2/df = 2.04, CFI = 0.944, TLI = 0.905, and RMSEA = 0.090. The findings revealed that the 95% CI for the mediating effect ranged from -0.506 to -0.109, without a value of 0 within the interval. There was a significant mediating effect of fertility stress on the relationship between irrational parenthood cognitions and social support, with the 95% CI for the direct effect ranging from -0.484 to 0.054; the interval contained 0, indicating no direct effect of social support on irrational parenthood cognitions. Details are depicted in Fig. 1.

## Discussion

A cross-sectional survey of 300 women from an Iranian infertility clinic by Laya Farzadi [19] revealed that the mean irrational parenthood cognition score was 39.7, which is similar to the results of this study, indicating that the surveyed patients with RIF had an internal desire to have a child to live a happy life. The outcomes of the cross-sectional study are comparable to those of the current study. In the findings of Zhao Jing [20] et al.’s study of factors related to morbidity stigma and infertility-related stress among 233 female infertility patients at the Wuhan Reproductive Centre, the mean infertility-related stress score was 137.69±29.42, which closely matched the mean total fertility stress score in the study by Newton et al. [21]; the fertility stress score of the patients with RIF in this study was considerably greater than those of the previous studies. The treatment of infertility is a process that involves both hope and disappointment; most patients are middle-aged and young adults who bear greater responsibilities and experience family- and career-related stress. This stress, coupled with repeated treatment cycles in the face of multiple implantation failures and high psychological fluctuations, significantly increases the level of fertility stress. Patients with an education level of high school or less and an irrational parenthood cognition score in the upper middle range had higher fertility stress scores and received relatively less social support. The higher a woman’s education level was, the lower the level of infertility stress. This may also be because the perceptions of fertility in patients with lower education levels are largely inherited from the traditional concept of women continuing the family line; therefore, these patients, who have a limited understanding of infertility and its treatment, are more likely to blame themselves for their inability to conceive. Additionally, they may be more affected by social opinions and prejudices, causing them to experience greater fertility stress. In contrast, patients with a higher degree of education can learn more pertinent knowledge and are better able to communicate with health care experts; consequently, they experience less fertility stress. The higher the annual household income level and the lower the levels of irrational cognitions and fertility stress are, the greater the level of social assistance received by patients. In women with regular jobs or high annual household income levels, work can be considered an accomplishment and balance the focus on fertility, and a higher household economic level ensures a higher quality of life, resulting in a relatively low perception that this group of infertility patients must have a child. This study also identified statistically significant differences between the irrational parenthood cognition scores of women with RIF and the relationship and social support scores of their partners. The degree of rationality of women's beliefs about fertility was associated with the quality of the couples’ relationships, with those who had better relationships with their partners having a greater degree of rationality about fertility and obtaining greater amounts of social support. This suggests that a healthy couple relationship and the support of one’s partner are particularly important in the treatment of female infertility, which does not improve somatic symptoms but significantly improves negative emotions in female patients, allowing them to face treatment with optimism and increase their resilience.

In this study, the greater the irrational parenthood cognition score was, the higher the fertility stress score; conversely, the higher the amount of social support, the more rational the perception of fertility. When enduring stressful events, irrational parenthood cognitions can lead to significant emotional and behavioral disturbances, whereas correct perceptions about infertility can lead to increased treatment cooperation and reasonable thinking about the stigma of the condition [22]. Although many cultures do not have specific solutions to childlessness, people do not accept childlessness passively [7]. Female patients with high levels of irrational parenthood cognitions believe that childlessness implies the loss of the parental role, making them deficient regarding typical family life and prone to increased marital conflict and crises [23]. Traditional perspectives on sexuality consider that limited or diminished reproductive potential indicates low sexual functioning, which impacts the gender role identities of couples experiencing infertility and causes a certain amount of sexual stress [24]. Some African cultures view infertility as an exclusive female problem [7]. A study of involuntary childlessness in seventy-eight societies found that women were significantly more likely to be blamed for childlessness than men, a fact attributed to the ubiquity of women’s low social statuses across cultures [25]. Infertility occurs in close to equal amounts for men and women, yet the general awareness of male factors contributing to involuntary childlessness is limited in society [26]. Almeling examined the reporting and dissemination of pregnancy and reproductive health information in the US cultural environment and found a lack of information on male reproductive health in the public domain [27]. This invariably increases the social and reproductive pressure on women with infertility, making them reluctant to interact with others and vulnerable to discrimination and prejudice, which can lead to inferiority complexes. Simultaneously, psychological problems caused by irrational fertility perceptions can have a negative impact on the medical behavior of women with infertility, thereby delaying the treatment process and resulting in a reluctance to communicate with others about fertility, decreasing the social support these patients receive. Therefore, during clinical therapy for infertility, we should have a comprehensive understanding of the lives and cultural backgrounds of such patients, urge them to actively seek and accept social support from all available sources, and provide them with professional health education on infertility. Assisting patients in replacing irrational ways of thinking and beliefs with rational ones can also be achieved through psychological interventions such as positive thinking therapy, group cognitive therapy, and rational emotive behavior therapy.

Figure 1 depicts the findings of the analysis of the mediating influence of reproductive stress on the relationship between irrational parenthood cognitions and social support in patients with RIF. However, social support did not have a direct effect on irrational parenthood cognitions. Specifically, the amount of social support received by women with RIF could not directly predict illogical parenthood cognitions, while the level of fertility stress could indirectly predict irrational parenthood cognitions. This may be because patients with RIF have experienced multiple IVF-ET failures and have expended a greater amount of human, material, and financial resources than other patients, creating strong psychological feelings of guilt, which manifest as social stress, couple relationship stress, sexual stress, and childlessness trade-off stress, hence predicting the level of illogical parenthood cognitions among female patients with RIF. The vast majority of patients undergoing assisted reproductive treatment aim to achieve a successful pregnancy and improve their quality of life and well-being. However, repeated failed transfers not only fail to meet the therapeutic requirement for a successful pregnancy but also increase the financial and psychological burdens on patients and their families, with familial and social pressures dominating the situation. Patients experience negative emotions such as low self-esteem, anxiety, and guilt due to infertility. This, combined with traditional thinking and previous transfer failures, depletes not only their savings but also increases the pressure these patients feel from their spouses and other sources, causing them to become even more nervous, anxious, fearful, and desperate about having children.

## Conclusions

Although children are both desired and expected in all cultures, the meanings attached to children and parenthood, the perceptions of infertility, sexuality, and medicine, and the nature of communication about these issues vary widely from culture to culture. Because of frequent implantation failures, patients cannot maintain their internal equilibrium and must face their friends, family, and relatives, resulting in negative feelings and, in some instances, social issues. Given the mobility of the population in today’s society, it is common to for physicians to treat patients from other cultures. To provide a better service, it is important to address not only medical issues but also to understand the social and psychological issues that may be affecting patients. Medical and nursing staff must thoroughly understand the cultural background, the couple relationship, and the psychological needs of patients and their families before initiating the treatment cycle, screen the fertility perceptions of women with infertility, and develop effective intervention strategies from the perspective of fertility stress, social support, and cultural background. Patients and their families can try to accept the belief that having children is not the only purpose of life [28], thus reducing the fertility stress of women with RIF. As the medical records included in this study were taken from a local study with a limited sample size, the sample size needs to be further expanded, and a multicenter study will be conducted in the future to further enhance the reliability of the findings.
